# Reverse left ventricular remodeling is more likely in non ischemic cardiomyopathy patients upgraded to biventricular stimulation after chronic right ventricular pacing

**DOI:** 10.1186/1476-7120-9-41

**Published:** 2011-12-16

**Authors:** Maria-Aurora Morales, Umberto Startari, Giuseppe Rossi, Luca Panchetti, Andrea Rossi, Marcello Piacenti

**Affiliations:** 1 CNR Clinical Physiology Institute, via G Moruzzi 1, 56124 Pisa, Italy; 2 Fondazione Toscana Gabriele Monasterio, via G Moruzzi 1, 56124 Pisa, Italy

**Keywords:** congestive heart failure, biventricular stimulation, non-ischemic cardiomyopathy, ischemic cardiomyopathy

## Abstract

**Background:**

Chronic right ventricular (RV) apical pacing may lead to left ventricular (LV) dyssynchrony and LV dysfunction. In heart failure due to RV pacing, upgrading to biventricular stimulation (CRT) can improve NYHA Class and LV function. A proportion of patients do not respond to upgrading. Aim was to assess whether etiology of LV dysfunction accounts for responses to CRT in RV-paced patients.

**Methods:**

Sixty-two patients treated by CRT, under RV pacing from 50.2 ± 5.4 months, were studied. Cause of LV dysfunction was non-ischemic (NIC) in 28 and ischemic cardiomyopathy (IC) in 34 patients. Clinical and conventional echocardiographic parameters were available within 1 month before RV pacing, within 1 month before CRT and at 12 ± 2 months of follow-up (FU).

**Results:**

Decreased LVEF (from 37.0 ± 8.8 to 25.6 ± 6.1%, p <0.001), increased LV end-systolic dimensions (LVESD) (from 48.1 ± 8.6 to 55.2 ± 7.9 mm, p <0.001) and worsened NYHA Class (from 1.9 ± 1.1 to 3.2 ± .6, p < 0.005) were found before CRT, compared to pre RV-pacing. After CRT, 44/62 patients showed a ≥ 1 NYHA Class improvement; >10% decrease in LVESD was observed in 24 patients: 5 with IC, 19 with NIC (p < .0.001). The association between cause of LV dysfunction with >10% decrease in LVESD remained highly significant (p < 0.001) adjusting for pre-CRT QRS duration, NYHA Class, LVEF, LVESD, treatment or RV pacing duration.

**Conclusions:**

CRT improves functional class even after long-lasting pacing. Reverse remodeling is evident in a small population, more likely with NIC.

## Background

Cardiac resynchronization therapy (CRT) is an established treatment for patients with drug refractory heart failure (HF) and left bundle branch block (LBBB) since it improves symptoms, quality of life and functional capacity [1,2], leading to reduction of hospitalizations for heart failure and death [3,4].

Like native left bundle branch block (LBBB), right apical ventricular (RV) pacing may result in intra- and interventricular dyssynchrony and development of LV remodeling due to an abnormal sequence of left ventricular activation [5-11].

Upgrading to CRT in RV paced patients has been shown to determine symptomatic and functional improvement comparable to that observed in non-paced LBBB patients [12-15]. Upgrading may also induce significant reverse remodeling in pacemaker-dependent patients [15]. However, until now, the clinical settings and conditions for upgrading conventional RV pacing to resynchronization have not been defined [16]: a percentage of patients still do not respond to upgrading, and conflicting results are reported on the role of underlying pathology in response to CRT in RV apical-paced patients [14,15].

In patients with ischemic cardiomyopathy CRT leads to a less significant improvement in left ventricular ejection fraction and reduction in left ventricular end-systolic volume than in patients with non-ischemic disease at mid-and long-term follow-up [17,18].

Therefore, the aim of this study was to assess whether the underlying cause of LV dysfunction may influence CRT-induced LV reverse remodeling in patients already under conventional RV apical pacing.

## Methods

Patient population. We retrospectively studied 62 consecutive patients admitted to our Institute for upgrading to biventricular pacing from April 2007 to December 2009 in sinus rhythm or atrial paced rhythm. Criteria for implantation were represented by NYHA Class =>III and LVEF < 35% under optimal medical therapy. Among patients with history of ischemic heart disease, only those with no active ischemia, assessed by either myocardial scintigraphy or dypiridamole echocardiography within 2 weeks before LV lead implant, and who had no indications to coronary revascularization, were enrolled in the study.

Fifty-one were males, mean age 73 ± 1.0 years; all patients were under continuous RV apical pacing for 51.2 ± 38.9 months (range 3 to 170 months), with mean QRS duration 180 ± 20 msec. At time of hospital admission patients were in NYHA Class III (n. 38) or IV (n.14) and were under optimal therapy with diuretics, ACE inhibitors or ATII blockers and beta blockers at maximal tolerated dosages before upgrading. Forty-eight patients were also under antialdosterone treatment.

Before upgrading, patients were defined as having ischemic cardiomyopathy (IC) if they had a history of myocardial infarction, and/or a history of coronary artery by-pass graft and/or angioplasty and/or a coronary angiogram indicating major disease. Patients were diagnosed as having non ischemic cardiomyopathy when no coronary artery disease could be detected by coronary angiography, significant valvular disease by conventional echocardiography and there was no documentation of myocarditis in their clinical history. On this basis the underlying cause of LV dysfunction was non-ischemic cardiomyopathy (NIC) in 28 and IC in 34 patients.

Seventeen patients had an implantable cardioverter defibrillator (ICD) that was upgraded to a CRT device, 35 patients had a pacemaker upgraded to CRT-Pace maker and 10 to CRT-Defibrillator.

LV leads were implanted by a transvenous approach through the coronary sinus and positioned in a posterolateral, lateral or anterolateral cardiac vein. LV lead position was assessed by an experienced radiologist and cardiologist in all patients before hospital discharge. In all patients the devices were programmed in rate responsive dual-chamber mode, and atrio-ventricular delay was optimized before discharge by a previously published method which takes into account the Doppler derived dP/dt by the mitral regurgitation jet [19].

### Echocardiographic examination

A standard transthoracic echocardiogram was performed by commercially available instruments (Acuson Sequoia, Acuson Corporation, Mountain View, CA, USA; Vivid system 7, GE/Vingmed, Milwakee, WI, USA). LV end-diastolic dimensions (LVEDD) and LV end-systolic dimensions (LVESD) were obtained from M-mode recordings derived from 2D echocardiograms of the LV transverse axis. These were obtained using the parasternal long axis approach with the M-mode cursor positioned at the tips of the mitral valve leaflets, using the leading-edge methods, according to recommendations of the American Society of Echocardiography. LV end-diastolic and systolic volumes and derived ejection fraction were assessed by the biplane summation method from the apical 4-chamber view [20].

Clinical and functional parameters by 2D echocardiography were available in all patients in three conditions: 1) within 1 month before first implant of RV pacing, 2) within 1 month before implantation of the LV lead and 3) at a mean of 12 ± 4 months follow-up (FU) after CRT. Before CRT, wall motion score index was also calcutated in all patients and mitral regurgitation severity determined semiquantitatively from color Doppler images obtained from the conventional parasternal long axis and apical views using the regurgitant jet area to LA area ratio [21]. The following score was used: 1: mild regurgitation, 2: moderate and 3: severe insufficiency. All echocardiograms were recorded and stored for off-line analysis by two reviewers blind to patient name and date of examination.

The investigation conforms with the principles outlined in the Declaration of Helsinki (Br 6 Med J 1964, ii:177).

### Statistical analysis

Results are presented as means ± SD. Changes in continuous variables before and after procedures were compared using paired Student's t-test. Analysis of variance was performed for comparing data between patients with NIC or IC and between patients with and without reverse remodeling. The chi-square test was used to compare categorical variables. A multiple logistic regression was used to assess the association between etiology (NIC or IC) and LV remodeling, categorized as >10% or < = 10% decrease in LVESD, adjusted for clinical and instrumental covariates. A p-value < 0.05 was considered statistically significant.

## Results

Before conventional PM implantation 22 patients were in NYHA Class I, 35 in NYHA Class II and 5 in NYHA Class III. Mean LVEF was 37.0 ± 8.9% (range 18 to 60%). Normal LV function at 2D Echo (LVEF >or = 50%) was reported in 12 patients. All pre conventional PM implantation parameters were extracted by reviewing hospital charts of the studied population.

**Figure 1:**
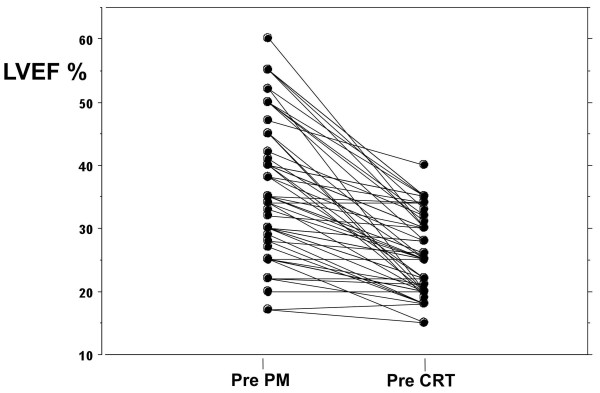
**Left ventricular ejection fraction (LVEF%) changes from pre conventional pacemaker implantation [pre PM] to pre upgrading [pre CRT] in the studied population**.

At time of CRT implantation, average right ventricular stimulation was > 90% (range 85 to 99%). When pre-upgrading data were compared to pre-conventional pace maker (PM) implantation, a significant worsening in NYHA Class (from 1.9 ± 1.1 to 3.2 ± .6, p < 0.005) along with a decrease in LVEF (from 37.0 ± 8.9 to 25.6 ± 6.1%, p <0.001), increase in LVEDD (from 60.9 ± 6.6 to 65.2 ± 7.7 mm, p <0.001) and LVESD (from 48.1 ± 8.6 to 55.2 ± 7.9 mm, p <0.001) were found in the studied population. In the subgroup of patients with a normal LV function before conventional PM implantation LVEF changed from 52.8 + 3 to 29.6 + 6 (p < 0.0001). In Figure 1 changes in LVEF for each studied patient from pre conventional PM implantation to pre CRT are reported. No relation could be reported between decreased LVEF and the duration of RV apical pacing at time of CRT. Twenty-six patients had a history of myocardial infarction before conventional PM implantation. However, none of the patients had documentation of myocardial infarction or acute coronary syndrome requiring hospitalization before upgrading.

**Figure 2:**
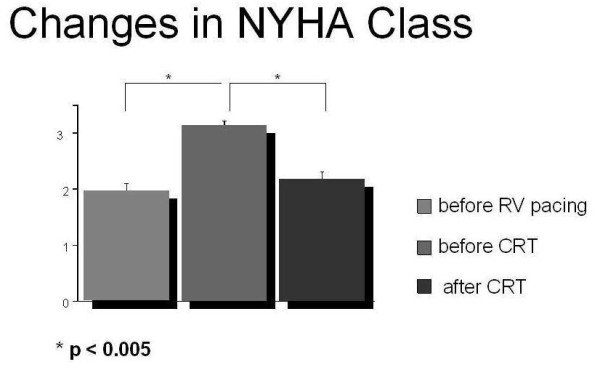
**NYHA Class data in the studied patients before RV implantation, before CRT and at FU are reported in the figure**.

At FU after CRT, NYHA Class decreased to 2.3 ± 0.5; a ≥ 1 NYHA Class improvement was seen in 44/62 patients, 19 with IC and 25 with NIC (p < 0.005). Figure 2.

No myocardial ischemic events from the time of CRT upgrade to the time of FU was documented in this subset of patients.

In the overall population QRS duration decreased from 180 ± 20 to 114 ± 11 msec (p < 0.01). No relation between QRS duration changes, NYHA Class and LVEF increase could be reported in the studied group.

At FU LVEDD changed from 65.2 ± 7.6 to 63.3 ± 7.9 mm (p < 0.1), LVESD from 55.2 ± 7.9 to 51.2 ± 8.4 mm (p < 0.001) and LVEF increased from 25.6 ± 6.1 to 31.4 ± 9.1% (p < 0.001). However, a >10% decrease in LVESD, as an index of consistent LV reverse remodeling after CRT, was observed in 24 patients only.

In table 1 the characteristics of all patients and those with and without reverse remodeling are reported. The only significant difference between the two groups was reported for duration of RV pacing (73 vs 37 months in pts with and without reverse LV remodeling, respectively, p < 0.01).

**Table 1:** Clinical and echocardiographic characteristics before upgrading of all patients and of those with or without documented >10% reduction in LVESD after CRT

	Total population (n.62)	Reverse remodeling (n.24)	No reverse remodeling (n.38)	p
Age (years)	73 ± 7.8	73.4 ± 8.2	72.6 ± 7.6	NS
NYHA Class	3.2 ± 0.6	3.2 ± 0.4	3.2 ± 0.5	NS
LVEF (%)	25.6 ± 6.1	26.4 ± 6.3	25.2 ± 6.0	NS
LVEDD (mm)	65.2 ± 7.7	65.9 ± 8.1	64.8 ± 7.6	NS
LVESD (mm)	55.2 ± 7.9	56.4 ± 8.4	54.4 ± 7.6	NS
Months of RV pacing	51.2 ± 38.9	73.2 ± 57.4	37.3 ± 38.2 *	P < 0.01
ACE-I or ARB (%)	85	84	86	
Beta blocker (%)	90	91	89	
Diuretics (%)	92	91	93	

As far as mitral regurgitation, the scores calculated before CRT and at follow up changed from 2.3 ± .8 to 2.1 ± .8, NS; however, when groups were divided according to >10% decrease in LVESD, a significant reduction could be reported in the group with reverse remodeling (from 2.2 ± .6 to 1.6 ± .6, p < 0.05) as compared to patients with no significant changes in LVESD (from 2.4 ± .8 to 2.4 ± .7).

**Figure 3:**
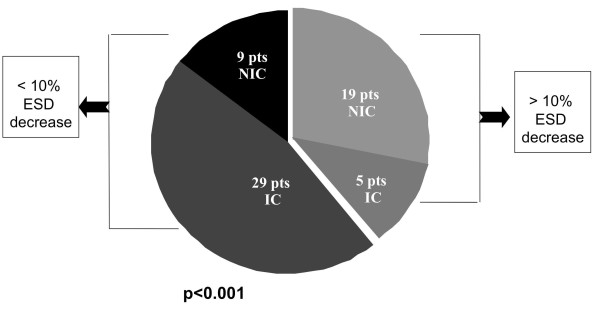
**Distribution of patients according to etiology of LV dysfunction and changes in end systolic dimensions (ESD) at FU**. IC: ischemic cardiomyopathy; NIC: non ischemic cardiomyopathy.

In terms of etiology of LV dysfunction, 5/34 patients with IC showed a >10% decrease in LVESD compared to 19/28 NIC patients (p < 0.001). Figure 3.

No differences in pre-CRT QRS duration, NYHA Class, LVEF, LVEDD, LVESD, ongoing treatment, site of LV lead implant, or duration of RV pacing could be observed between the 28 NIC patients vs the 34 IC patients (Table 2). Patients with NIC had a slightly, although not significant lower WMSI before upgrading, when compared to the IC patients (2.15 ± 2.1 vs 2.26 ± 2.3).

**Table 2:** Clinical and echocardiographic characteristics before upgrading of patients with IC or NIC

	IC (n.28)	NIC (n.34)	p
Age (years)	70.1 ± 8.0	74.5 ± 7.3	NS
NYHA Class	3.3 ± 0.5	3.2 ± 0.4	NS
LVEF (%)	26.0 ± 6.7	25.3 ± 5.7	NS
LVEDD (mm)	66.9 ± 8.0	63.8 ± 7.3	NS
LVESD (mm)	56.7 ± 9.1	54.0 ± 6.7	NS
Months of RV pacing	51.4 ± 42.4	47.5 ± 40.9	NS

LVEF: left ventricular ejection fraction, LVEDD: left ventricular end diastolic dimensions, LVESD: left ventricular end systolic dimensions. RV: right ventricular

In a multivariate analysis by multiple logistic regression, the association between cause of LV dysfunction with >10% LVESD decrease remained highly significant (p < 0.001) also adjusting for pre-CRT QRS duration, NYHA Class, LVEF, LVEDD, LVESD, ongoing treatment, site of LV lead implant, or duration of RV pacing. (Table 3).

**Table 3:** Predictors of reverse left ventricular remodeling at univariate and multivariate analysis by Cox model

Variables	Univariate HR (95% CI)	P value	Multivariate HR(95% CI)	P value
NIC	12.24 (3.55-42.18)	<0.001	26.27 (0.37-158)	<0.001
QRS duration	1.02 (0.99-1.04)	0.206	1.01 (0.97-1.05)	0.664
NYHA	0.99 (0.31-3.12)	0.985	1.16 (0.20-6.68)	0.867
Site of implant PL	0.69 (0.23-2.06)	0.686	0.48 (0.09-2.71)	0.412
Site of implant A	0.48 (0.08-3.03)	0.435	0.06 (0.004-1.03)	0.053
RV pacing (mo)	1.02 (1.004-1.03)	0.013	1.03 (1.01-1.05)	0.012
LVEF	1.03 (0.95-1.12)	0.438	1.19 (0.97-1.47)	0.101
LVEDD	1.02 (0.95-1.09)	0.589	0.76 (0.59-0.99)	0.044
LVESD	1.04 (0.97-1.11)	0.299	1.35 (1.01-1.82)	0.044

Legend: NIC: non ischemic cardiomyopathy. PL: postero-lateral. A: Anterior. Mo: months HR: hazard Ratio. See text for other abbreviations.

In the IC group, the 24 patients with documented previous MI had NYHA Class, LVEF and LVESD, WMSI at echocardiography, comparable to those obtained in the 10 patients without MI.

## Discussion

The results of this study confirm previously published data that RV apical pacing may lead to overall reduction in LV global function. Upgrading to CRT improved NYHA Class in over 70% of patients but a significant reduction in LVESD at a mean of 1 year FU was observed in the majority of patients with NIC, while it was less evident in IC patients.

RV apical pacing is associated with asynchronous electrical activation of the left ventricle which impairs cardiac systolic and diastolic function and induces regional perfusion defects even in the absence of coronary artery disease [22-25].

Upgrading to CRT may determine improvement in clinical symptoms, LV global function and reduces or abolishes dyssynchrony in chronically paced patients [12,13,16,26,27] leading to left ventricular reverse remodeling in a way similar to primary CRT [14,15].

Molhoek et al. demonstrated comparable benefits from primary CRT in patients with ischemic vs non-ischemic cardiomyopathy in terms of NYHA Class, quality of life, and LVEF [28]. However, larger studies have shown that NIC patients are more likely to improve clinically and functionally. In the PROSPECT study a greater decrease in LV end systolic volumes was documented in patients with non-ischemic HF when compared to those with an ischemic substrate [29]. Accordingly the MIRACLE and CARE-HF studies showed that reverse LV remodeling occurs to a lesser degree in patients with ischemic etiology [30,31]. Hyper-responders, defined as those who show complete functional recovery up to normalization of LV function after CRT are almost exclusively seen in non-ischemic cardiomyopathy [32,33]. From a pathophysiological point of view, large areas of myocardial scar in ischemic cardiomyopathy may determine complex patterns of LV activation that are not amenable of correction with CRT [34]. In addition, areas of the scar as occurring in post ischemic LV dysfunction may limit LV reverse remodeling after CRT. Therefore, it is understandable that nonischemic HF patients may show LV reverse remodeling to a larger extent than ischemic patients [35].

In this study clinical improvement was reported in a high percentage of patients, comparable to previously published data. Reverse LV remodeling could be found in only 24 patients; of these, 19 had a non-ischemic etiology of underlying LV dysfunction.

In heart failure patients, reversal of LV remodeling after pharmacologic treatment has important prognostic implications, as previously documented in the SOLVD study [36]. Prognosis after CRT was shown to be related to the extent of LV reverse remodeling, more than to clinical response [33,37,38]). The definition of LV reverse remodeling is highly variable in literature and changes in LV volumes or diameters have been considered by different AA [29-33]. In line with previously published papers from our group [39] we defined reverse remodelling as a >10% reduction in LVESD; low reproducibility and inaccuracy of the methods commonly used for volume calculations by 2D echocardiography are known, possibly due to the inadvertent use of foreshortened views of the left ventricle and the reliance on geometric modeling [40]. End systolic diameter measurement of is then more reliable, in particular in patients with ischemic dysfunction.

It is important to say that baseline characteristics in NIC and IC were comparable before CRT, in terms of treatment, QRS duration, PM implantation duration, baseline LV function. Interestingly, patients who had been under RV apical pacing for longer periods were those who showed the best functional recovery after upgrading. A possible explanation may be represented by the chronic detrimental effect on LV function provided by right ventricular pacing, which can be reverted by LV resynchronization. Tse et al [9], have shown that long periods of right ventricular pacing result in a high incidence of myocardial perfusion defects. It can be speculated that in these patients upgrading may abolish or attenuate the perfusion defects leading to left ventricular contractile improvement and reduction in end systolic dimensions. In patients in whom upgrading was performed after a short period of RV apical pacing due to clinical and functional worsening, the lack of LV reverse remodeling could have been attributed more to the natural history of the disease, and the correction of dyssynchrony unable to counteract the decline in overall LV function. Another interesting result is represented by the role of LVEDD and LVESD before implant: while LVEDD is a negative factor for LV remodeling after CRT, a bigger LVESD may be a predictor of recovery after upgrading. In a previously published paper, patients who were super responders (>30% reduction in end-systolic volume) and responders after de novo CRT were those showing bigger baseline end-systolic volumes, as compared to patients who functionally worsened at 6 months FU [33].

In conclusion, upgrading to CRT determines improvement in NYHA class in a good percentage of patients. Significant LV reverse remodeling is more likely to occur in patients with non-ischemic cardiomyopathy. Long FU are needed to identify with proper viability/contractility studies [41,42] patients who may really benefit from upgrading to CRT.

## Limitations

The main limitation of this study is that it is a single-center and retrospective cohort study. However clinical data after conventional PM implantation were collected systematically at each programmed out-patient check-up and echocardiograms were analyzed by two reviewers who were blinded to patient name and date of examination.

## Abbreviations

LV: left ventricle; RV: right ventricle; LVESD: LV end-systolic dimensions; LVEDD: LV end-diastolic dimensions; NIC: Non-ischemic cardiomyopathy; IC: Ischemic cardiomyopathy; WMSI wall motion score index; PL: Posterolateral; A: anterior.

## Competing interests

The authors declare that they have no competing interests.

## Authors' contributions

MAM Concept/design. US Data analysis. GR Statistics. LP Data collection. AR Data collection. MP Critical revision and approval of the article. All Authors read and approved the final manuscript.
